# Plantar Flexor Weakness and Pain Sensitivity Cannot Be Assumed in Midportion Achilles Tendinopathy

**DOI:** 10.1249/esm.0000000000000017

**Published:** 2023-10-26

**Authors:** Lauren K. Sara, Savannah B. Gutsch, Marie Hoeger Bement, Sandra K. Hunter

**Affiliations:** 1 Harvard Medical School, Cambridge, MA, USA; 2 Department of Physical Therapy, Marquette University, Milwaukee, WI, USA

**Keywords:** Contractile function, Contractility, Force-velocity, Neural drive, Pressure-pain threshold, Voluntary activation

## Abstract

**Introduction/Purpose:**

The purpose of this study was to determine the following in persons with midportion Achilles tendinopathy (AT): 1) maximal strength and power; 2) neural drive during maximal contractions and contractile function during electrically evoked resting contractions; and 3) whether pain, neural drive, and contractile mechanisms contribute to differences in maximal strength.

**Methods:**

Twenty-eight volunteers (14 AT, 14 controls) completed isometric, concentric, and eccentric maximal voluntary contractions (MVCs) of the plantar flexors in a Biodex^™^ dynamometer. Supramaximal electrical stimulation of the tibial nerve was performed to quantify neural drive and contractile properties of the plantar flexors. Pain sensitivity was quantified as the pressure-pain thresholds of the Achilles tendon, medial gastrocnemius, and upper trapezius.

**Results:**

There were no differences in plantar flexion strength or power between AT and controls (isometric MVC: *P* = 0.95; dynamic MVC: *P* = 0.99; power: *P* = 0.98), nor were there differences in neural drive and contractile function (*P* = 0.55 and *P* = 0.06, respectively). However, the mechanisms predicting maximal strength differed between groups: neural drive predicted maximal strength in controls (*P* = 0.02) and contractile function predicted maximal strength in AT (*P* = 0.001). Although pain did not mediate these relationships (i.e., between maximal strength and its contributing mechanisms), pressure-pain thresholds at the upper trapezius were higher in AT (*P* = 0.02), despite being similar at the calf (*P* = 0.24) and Achilles tendon (*P* = 0.40).

**Conclusions:**

There were no deficits in plantar flexion strength or power in persons with AT, whether evaluated isometrically, concentrically, or eccentrically. However, the mechanisms predicting maximal plantar flexor strength differed between groups, and systemic pain sensitivity was diminished in AT.

## INTRODUCTION

Midportion Achilles tendinopathy (AT) is an overuse injury of insidious onset resulting in pain and stiffness in the midportion of the Achilles tendon. Although commonly attributed to deficient plantar flexor muscle function (1,2), there is limited empirical evidence that supports this assumption, particularly compared with healthy controls. Instead, the premise of weak or fatigable plantar flexors in AT is largely based on two factors: 1) onset commonly accompanies a sudden increase in activity, implying that insufficient strength was a predisposing factor (2,3), and 2) strength gains often—but not always (4)—accompany improvements in symptoms and function (5).

According to a recent systematic review (6), the state of plantar flexor muscle function in AT is inconclusive; of six studies comparing AT with controls, three reported no difference (7–9), one reported impaired muscle function in AT (10), and two had mixed results within their respective cohorts (11,12). No study simultaneously evaluated isometric, concentric, and eccentric strength or the contractility of or ability to activate the plantar flexor muscles, limiting the ability to draw conclusions regarding maximal strength, mode of contraction, and contributing mechanisms. Despite existing data, the presumption of weakness in AT remains, as do recommendations for strengthening. However, strengthening interventions do not consistently resolve symptoms and functional limitations in AT (2,6). If strength is deficient, strengthening interventions would be expected to resolve the condition more reliably and consistently.

Weakness associated with impaired function, which might occur in AT, could be due to inadequate neural drive (i.e., the ability of the central nervous system to activate a muscle), muscle atrophy, or inability of the available muscle to contract. Neural drive and muscular contractility can be estimated using electrical stimulation (13). Voluntary activation (VA), a measure of neural drive, evaluates the ratio of torque generated during a maximal effort contraction to the torque generated when a muscle, motor nerve, or motor cortex is stimulated. The torque produced in response to electrical stimulation at rest, known as a resting twitch (RT), provides information about the force-generating capacity of the muscle or contractility, independent of nervous system activation. Understanding contributions of neural drive and muscular contractility to neuromuscular function in AT may help explain why symptoms are often unresolved with strengthening interventions alone.

Pain is another factor that can limit optimal muscle function. Activation of group III and IV motor afferent nerves, for example, can result in inhibition of motoneurons and reduce VA (14). Such somatosensory feedback is likely imperative to protect exercising muscle (15). However, in chronic pain populations, such inhibition could result in muscle atrophy and persistent abnormalities in neuromuscular function (16–18).

The purpose of this study was to determine the following in persons with AT: 1) maximal plantar flexion strength and power during isometric and dynamic contractions; 2) neural drive during maximal effort contractions and contractile function during electrically evoked contractions at rest; and 3) the contributions of pain, neural drive, and contractile mechanisms to maximal strength. Based on prevailing assumptions and possible interactions with pain, we hypothesized that people with AT would be weaker than controls because of inadequate neural drive.

## METHODS

### Participants

Twenty-eight volunteers participated in the study: 14 with AT and 14 controls (Table 1). AT inclusion criteria were history of gradual, insidious onset of pain and/or stiffness at the midportion of the Achilles tendon, which had become chronic (i.e., persisted for at least 3 months); pain reproduction with palpation of the midportion of the tendon; a positive Arc Sign; and a positive Royal London Hospital Test (2). Clinical assessments were completed as previously described (19). Controls were without history of either pain or stiffness in the Achilles tendon region. Exclusion criteria included diabetes (20); thyroid disorders (21); cardiovascular disease; neurological disease; known contraindications to exercise; and any acute injury, bursitis, insertional tendinopathy, or osteoarthritis in either lower extremity. Informed consent was obtained from all participants before participation in the study. The study protocol was approved by the Marquette University Institutional Review Board (HR-1801021327) and in compliance with the Declaration of Helsinki.

The study involved measures of maximal strength, pressure-pain thresholds (PPTs), VA, and RT of the plantar flexor muscles while seated in a Biodex^™^ dynamometer (Biodex System 3 Pro; Biodex Medical, Shirley, NY, USA). The following muscles have a moment arm and fiber direction that enables contribution to ankle plantar flexion: medial and lateral gastrocnemii (MG and LG, respectively), soleus (SOL), fibularis longus and brevis, posterior tibialis, flexor digitorum longus, and flexor hallucis longus. However, it is not possible to parcel out torque contributions of individual agonist muscles from the total plantar flexion torque measured during dynamometry. Thus, this study refers to “plantar flexors” rather than attributing maximal strength to any individual muscle or muscles. All testing sessions were led by the same researcher (L.K.S.). Testing took place during two sessions, and all tests were completed for both lower extremities. The order of limbs was randomized every session. Within sessions, testing was completedon one leg before performing identical testing of the contralateral limb.

The first session involved 1) familiarization to plantar flexor strength testing in the dynamometer, electrical stimulation of the tibial nerve (for VA and RT), and PPTs using a Somedic algometer (Somedic SenseLab AB, Sösdala, Sweden); 2) clinical measurements; and 3) completion of self-report outcome measures, including the Tampa Scale of Kinesiophobia (22), the Foot and Ankle Ability Measure (FAAM) (23), and the Victoria Institute of Sport Assessment—Achilles Questionnaire (VISA-A) (24), which evaluate fear of pain or injury, symptoms or difficulty with several functional tasks and activities of daily living, and the effect of AT on participation in activities. The second session involved evaluation of isometric MVCs with electrical stimulation, concentric and eccentric MVCs, baseline PPTs, and body composition measurements (using dual x-ray absorptiometry). Muscle activity of the triceps surae and tibialis anterior (TA) muscles was measured using surface electromyography (EMG). Physical activity data were calculated based on responses to the 12-month, self-report Modifiable Activity Questionnaire (25).

### Experimental Setup

**Figure 1.**
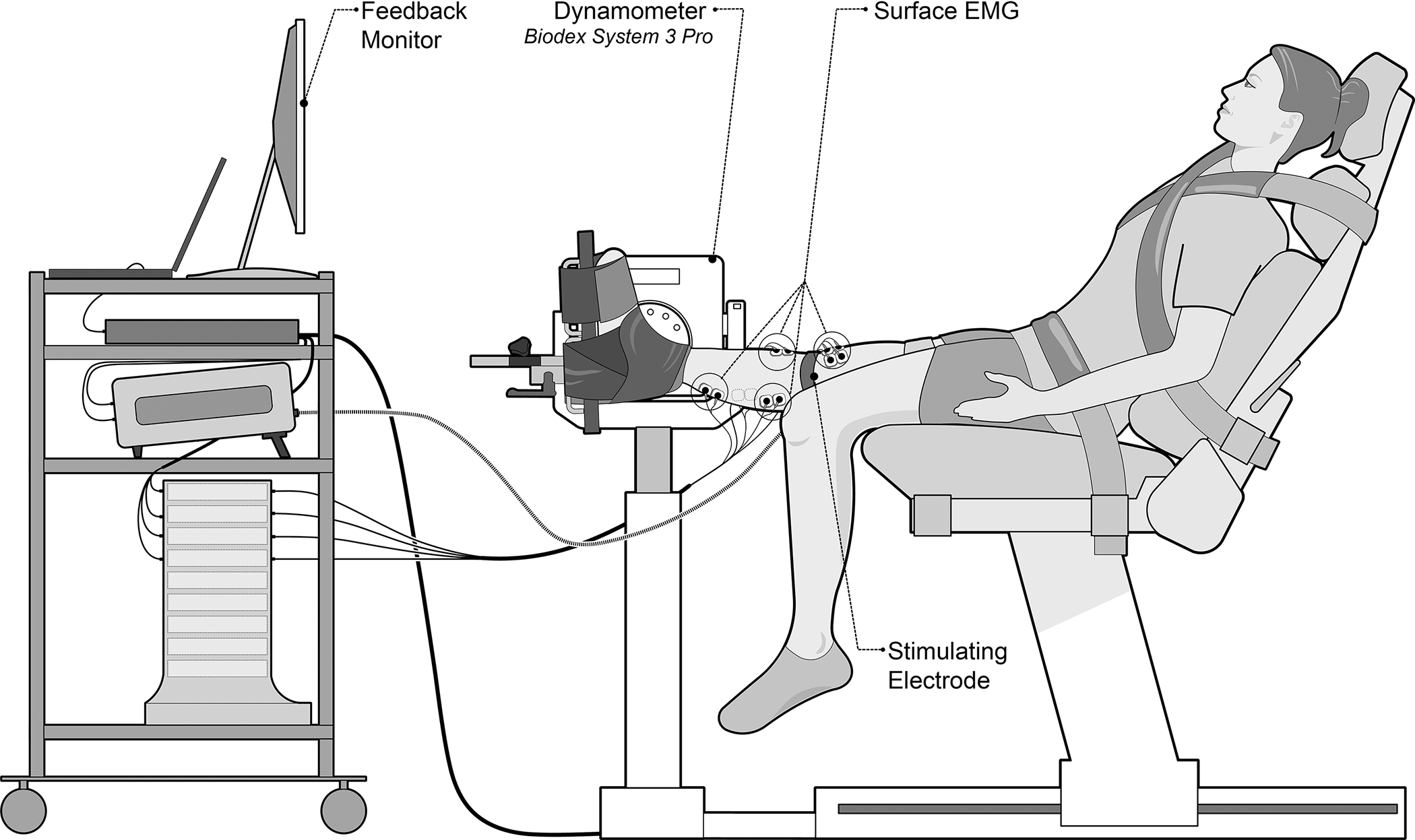
Experimental setup of a participant in the Biodex System3 Pro dynamometer used for plantar flexor strength measurements. Participants were given visual feedback on a monitor. Surface electromyography (EMG) was recorded for the medial and lateral gastrocnemii, soleus, and anterior tibialis muscles. Electrical stimulation was positioned over the tibial nerve.

Each participant was seated in the dynamometer with a straight knee (0° flexion) and a trunk angle of 55° (to minimize hamstring discomfort and sciatic nerve tension during testing). The thigh rested on a padded thigh support, and the foot rested on a foot plate affixed to the dynamometer (Fig. 1). To minimize extraneous movement and isolate exercise to the ankle joint, straps were placed across the waist, chest, and thigh. Two straps were secured around the ankle and one around the forefoot to maintain the plantar surface of the foot in contact with the foot plate. For PPT measurements, the ankle straps were removed to access the Achilles tendon, and the ankle was placed in a neutral position (i.e., foot perpendicular to shank).

To assess muscle activity in response to electrical stimulation, EMG electrodes were placed on the SOL, MG, LG, and TA muscles of bilateral lower extremities in a bipolar configuration (Ag–AgCl, 8-mm diameter; 20-mm interelectrode distance; Natus Medical Inc., Middleton, WI, USA) in accordance with recommendations from the Surface Electromyography for the Non-Invasive Assessment of Muscles project (26). Data were amplified (4000 Hz; Coulbourn Instruments, Allentown, PA, USA), digitized, and stored online (Power1401, Spike2 software; Cambridge Electronic Design Limited, Cambridge, UK).

### Pressure-Pain Thresholds

PPTs were measured with the participant seated in the dynamometer using a 1-cm^2^ algometer probe tip aligned perpendicular to the tissue being tested and at an application rate of 30 kPa·s^−1^ (figure, Supplemental Content 1, http://links.lww.com/EM9/A15). Algometry is a reliable method for evaluating PPTs within and between sessions (27,28). PPT was defined as the minimum pressure required to induce pain (28). Evaluation of PPTs local and remote to injured tissue provides valuable information about peripheral and central mechanisms, respectively, and is recommended for inclusion in tendinopathy populations (29,30).

PPT familiarization included emphasis that the measurement was not a test of pain *tolerance* but rather of the point at which the pressure sensation was first perceived as painful. Measurements were completed at three sites: 1) the Achilles tendon, using the pinch handle 4 cm proximal to the calcaneal insertion; 2) the ipsilateral MG, midway between lateral and medial margins at the location of largest calf girth; and 3) the upper trapezius, along its superior margin halfway between the seventh cervical vertebrae and the acromion process (see “x” marks in the figure, Supplemental Content 1, http://links.lww.com/EM9/A15). Participants pressed a button when the pressure sensation was first perceived as painful. Two measurements were completed at each measurement site, then averaged in subsequent analyses. Measurement order was randomized for each leg, participant, and session.

### Isometric Strength, VA, and Contractile Properties

Isometric strength was assessed using isometric maximal voluntary contractions (MVCs), VA was assessed with the interpolated twitch technique, and RT was evaluated in response to electrical stimulation of the resting muscle in a potentiated state. Torques produced during doublet stimulation were used in analyses of VA and RT.

To ensure supramaximal stimulation, isometric MVCs with electrical stimulation were assessed after determining optimal stimulation levels (31). In brief, the ipsilateral tibial nerve was stimulated with a bar electrode and a constant-current, variable high-voltage stimulator (DS7AH; Digitimer Ltd, Hertforshire, UK). Stimulation was applied at the medial popliteal space distal to the sciatic nerve bifurcation.Single square-wave pulses (400 V, 100 μs duration) were delivered with a stimulation intensity initiated at 50 mA and gradually increased until torque and EMG responses were optimized. The intensity was increased an additional 10% to ensure supramaximal stimulation during RT and VA.

To determine VA, the triceps surae muscles were electrically stimulated during an isometric MVC and then immediately after the MVC (~2 s after), during the muscles’ potentiated state. VA was calculated as the ratio of torque produced in response to doublet stimulation during isometric MVC (known as a superimposed twitch; SIT) to the torque produced in response to doublet stimulation at rest, while in its potentiated state (RT) (13): VA = 100 × (1 − SIT/RT). Immediately after resting doublet stimulation, a single-pulse stimulation was completed to evaluate compound muscle action potentials. Doublet stimulations were used for calculating VA, because they have been shown to be more sensitive than single stimulations (32). Participants completed four isometric MVCs, each separated by 2 min of rest.

The following contractile properties from the singlet stimulation were evaluated: rates of torque development and relaxation; maximum peak-to-peak compound muscle action potentials (Mmax; a measure of muscle activity in response to electrical stimulation) as measured using EMG of the triceps surae muscles; and electromechanical delay, defined as the time between onset of EMG activity and plantar flexion torque (33).

### Dynamic Strength

Maximal dynamic strength was assessed using concentric and eccentric MVCs at eight velocities, in increments of 30 from −90 to 150 deg·s^−1^ (negative velocities indicate eccentric activation). Velocities faster than 150 and −90 deg·s^−1^ were not utilized; during pilot testing, most healthy participants could not reach target velocities greater than 150 deg·s^−1^ within their available ankle range of motion, and eccentric torques plateaued by −90 deg·s^−1^.

Concentric and eccentric MVCs were completed using each participant’s available range of motion. Familiarization included four repetitions of both concentric and eccentric MVCs at 60 deg·s^−1^.Test contractions included four repetitions of maximal-effort contractions at each of the eight velocities, whose order was randomized per limb and participant. Participants rested 1 min between each set of MVCs. Strong verbal encouragement and visual feedback were provided for every MVC in the study, including familiarization.

### Data and Statistical Analyses

The best of four torques at each testing velocity were used for analyses. Isometric MVC was measured as the average of a 0.5-s window surrounding the maximum torque but preceding the superimposed electrical stimulation. Root-mean-squared EMG was recorded during the same 0.5-s window. Concentric and eccentric MVCs were measured as the instantaneous peak torque during dynamic contractions. Peak power was calculated as the product of instantaneous peak torque and velocity at time of peak torque. Rates of torque development and relaxation were normalized to isometric MVC torque. Mmax was normalized to the root-mean-squared EMG recorded during the 0.5-s window corresponding to peak isometric MVC torque.

Independent samples *t*-tests were used to compare physical characteristics, physical activity levels, and kinesiophobia between groups. Multivariate analyses of covariance (ANCOVAs) were used to evaluate between-group effects of AT diagnosis on strength, VA, and RT, with covariates of biological sex and age. Mediation analyses were performed to evaluate the effects of AT on PPT and of PPT on strength, VA, and RT. Repeated-measures ANCOVAs were used to compare limbs in persons with AT using a similar approach: outcome variables of strength, VA, and RT, and mediation analysis using PPTs.

Regression analyses were completed to assess the contributions of RT, VA, and pain to isometric MVCs. Normality was assumed for all variables based on histograms and Q-Q plots. *A priori* significance was set to *P*<0.05. Data are reported as mean ± standard deviation (SD) in the text and table and displayed as mean ± standard error of the mean (SEM) in the figures. Analyses were performed using the Statistical Package for Social Sciences (SPSS, V26; IBM Corp., Armonk, NY, USA).

## RESULTS

Of the AT participants, half had bilateral AT. Thus, for within-group analyses, limbs were divided into more and less affected limbs and referred to, for simplicity, as “AT” and “AT–Control.” AT symptom duration ranged from 3 to 240 months. Limbs were matched for dominance when comparing AT and controls. Despite efforts to match based on age and sex, the AT group was older and had a greater proportion of male participants than the control group (Table 1). Consequently, age and sex were used as covariates in statistical analyses. Torque data were normalized to body weight.

Based on FAAM and VISA-A, pain and disability were greater in AT than control or AT–Control limbs (Table 1). Kinesiophobia did not differ between groups. Maximal AT pain occurred 2.9–5.4 cm proximal to the calcaneal notch.

Isometric MVC plantar flexion torque was not different between AT and controls when controlling for biological sex and age (*P* = 0.89; Table 1), including when normalized to body weight (*P* = 0.21; figure, Supplemental Content 2, http://links.lww.com/EM9/A16). Likewise, isometric torque was not different between AT and AT–Control limbs (*P* = 0.19).

**Figure 2.**
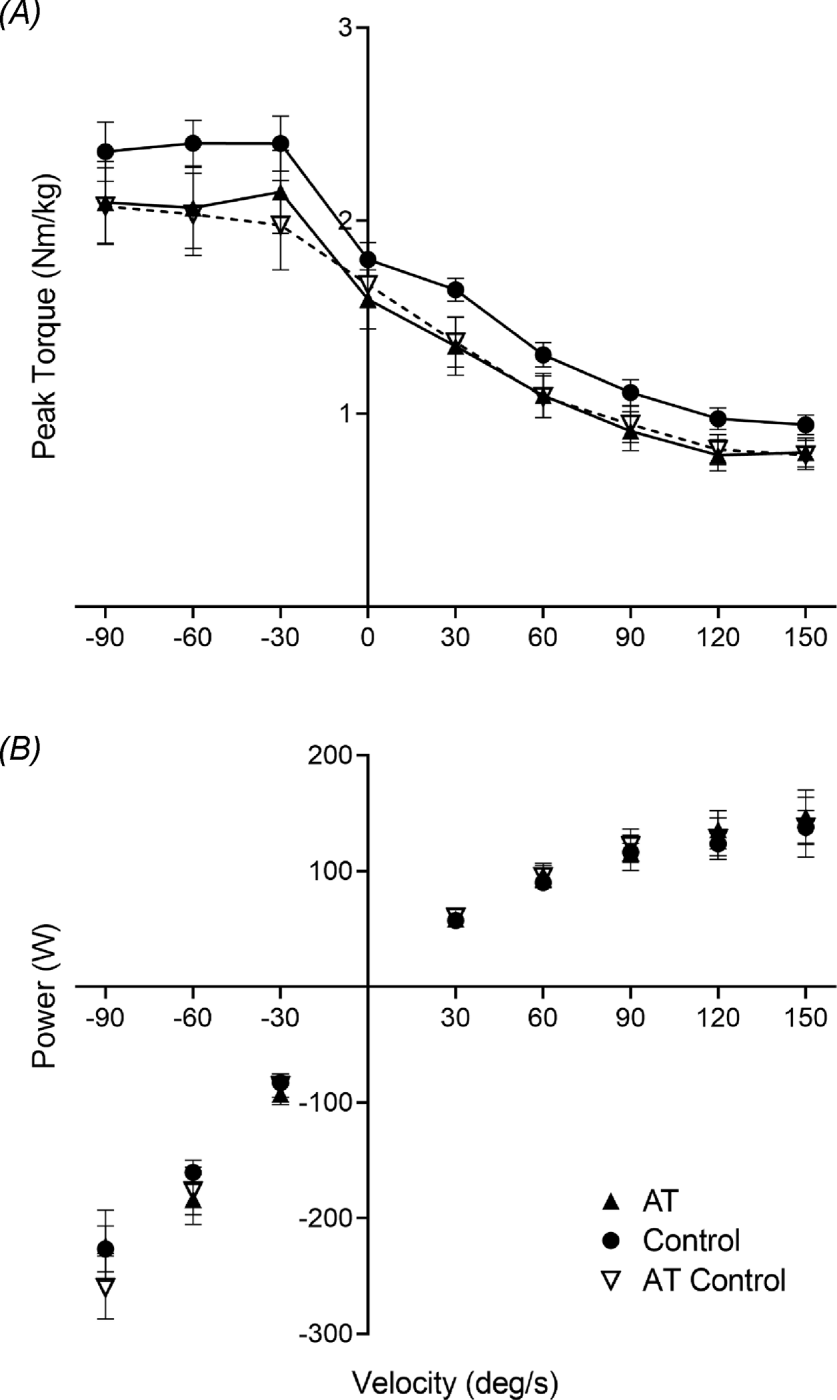
Plantar flexor torque–velocity and maximal power normalized to body weight. A, Plantar flexor torque–velocity relationship normalized to body weight. There were no differences between Achilles tendinopathy (AT) and healthy controls (*P* = 0.13) or between AT and AT–Control limbs (*P* = 0.60). B, Maximal plantar flexor power at each dynamic velocity normalized to body weight. There were no differences between AT and the matched-dominance limb in controls (*P* = 0.84) or between limbs in AT participants (*P* = 0.97).

Concentric and eccentric MVC torque and power were not different between groups at any velocity when controlling for age and sex, whether analyzed collectively across velocities (torque: *P* = 0.99; power: *P* =0.98) or at individual velocities (Fig. 2). There was a main effect of velocity for torque (*P* = 0.001) and power (*P* = 0.04). When comparing AT and AT–Control limbs, there were no differences in concentric and eccentric MVC torque (*P* = 0.88) or power (*P* = 0.40). When analyzed at individual velocities, the findings were similar: maximal dynamic (concentric and eccentric) plantar flexor strength was not different between AT limbs.

When controlling forage and sex, RT—a measure of contractile function—was similar in AT and controls (*P* = 0.07; Table 1). RT was not different between AT limbs (*P* = 0.37). There were no differences in VA between groups, controlling for age and sex (*P* = 0.53), or between limbs (*P* = 0.30; Table 1).

Consistent with doublet RT, there were no between-group differences in singlet RT (*P* = 0.41) rates of torque development (*P* = 0.92) or relaxation (*P* = 0.94), contraction time (*P* = 0.12), or half-relaxation time (*P* = 0.99). There were no AT between-limb differences in these variables (rate of torque development: *P* = 0.23; rate of torque relaxation: *P* = 0.22; contraction time: *P* = 0.86; half-relaxation time: *P* = 0.99; figure, Supplemental Content 3, http://links.lww.com/EM9/A17). Mmax was not different between groups (LG: *P* = 0.70, MG: *P* = 0.60; SOL: *P* = 0.95; TA: *P* = 0.51) or between limbs for any muscles (LG: *P* = 0.83, MG: *P* = 0.67; SOL: *P* = 0.64; TA: *P* = 0.79). There were no differences in electromechanical delay between groups (AT: 9.9 (2.7) ms, control: 10.9 (3.0) ms; *P* = 0.38) or between limbs (AT–Control: 9.5 (4.7) ms, *P* = 0.76). Current amplitudes did not differ between groups (AT: 310.6 (152.2) mA, control: 272.6 (99.1) mA; *P* = 0.44).

**Figure 3.**
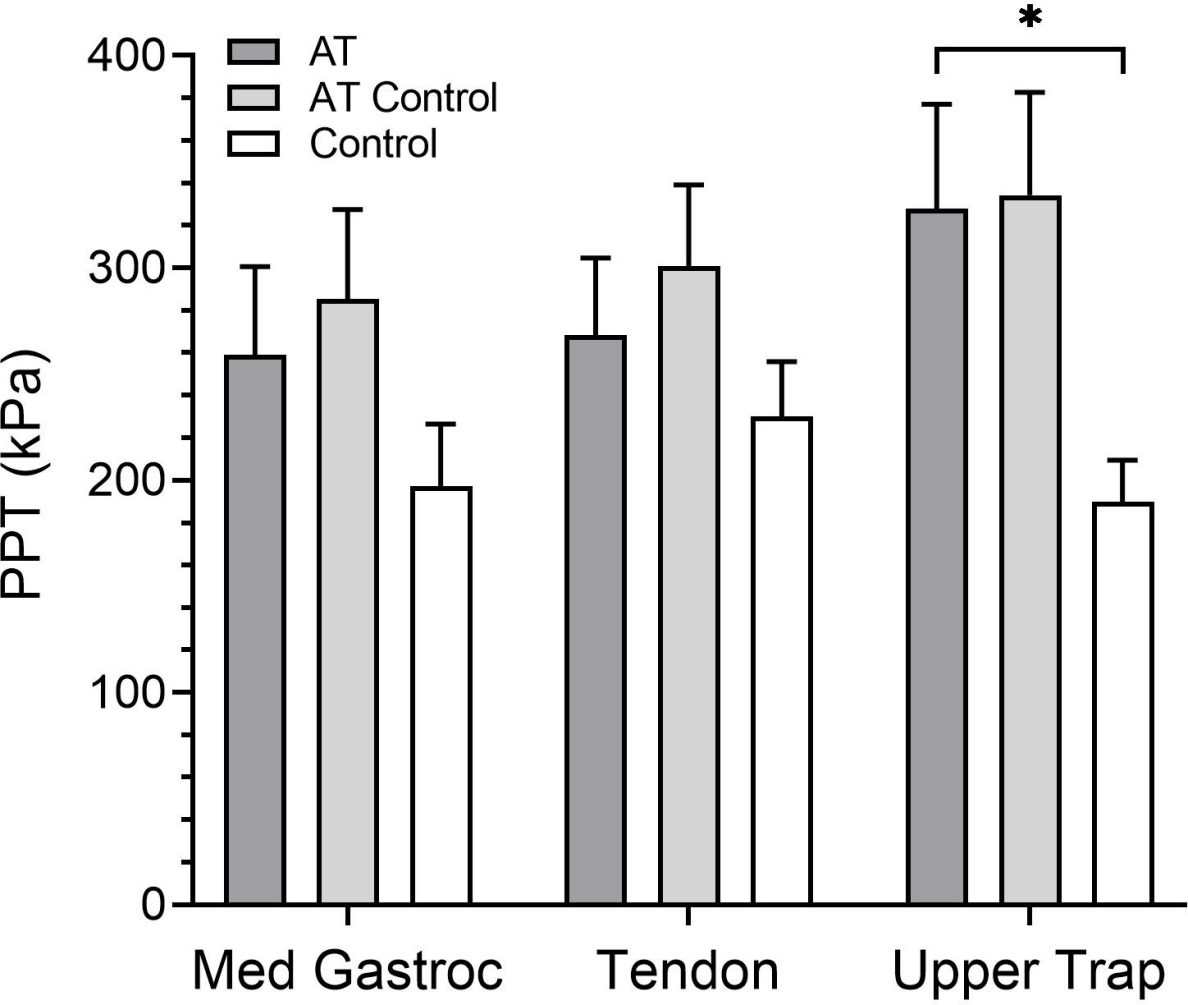
Pressure-pain thresholds (PPTs) at the medial gastrocnemius (Med Gastroc; MG), Achilles tendon, and upper trapezius (Upper Trap; UT) in Achilles tendinopathy (AT) compared with controls. MG and AT PPTs were not different between the more affected AT limb and the matched-dominance limb in controls (*P* = 0.12 and *P* = 0.20, respectively). UT PPTs were higher in AT compared with controls (*P* = 0.008). *P < 0.05.

Upper trapezius PPTs were higher in AT than controls (AT:327.86 (184.60) kPa, control: 189.82 (73.31) kPa; *P* = 0.02; Fig. 3). This difference remained significant adjusting for age (*P* = 0.02), sex (*P* = 0.03), or physical activity (*P* = 0.05). However, it was not significant when adjusting for body weight alone (*P* = 0.20) or for the combined variables of physical activity, age, sex, and body weight (*P* = 0.39). There were no differences in calf (AT: 258.86 (156.51) kPa, control: 197.14 (109.53) kPa; *P* = 0.24) or Achilles tendon PPTs (AT: 268.18 (136.78) kPa, control: 229.86 (97.26) kPa; *P* = 0.40; Fig. 3), even when controlling for sex, age, body weight, and physical activity (calf, *P* = 0.93; Achilles tendon, *P* = 0.65). AT and AT–Control limbs were not different at the upper trapezius (AT–Control: 334.25 (181.44), *P* = 0.93), calf (AT–Control: 285.21 (158.22), *P* = 0.66), or Achilles tendon (AT–Control: 300.82 (142.74), *P* = 0.54).

Predictors for isometric MVC strength included in the regression analysis were RT, VA, and upper trapezius PPT. Because Achilles tendon and calf PPTs did not differ between groups, only upper trapezius PPTs were included in this analysis. In controls, the set of predictors significantly contributed to isometric MVC (F(3,10) = 3.93, *P* = 0.04). However, when controlling for other predictors, VA was the only predictor of isometric MVC in controls (*t*(10) = 2.70, *P* = 0.02), and the proportion of variance uniquely explained by VA (${\text{sr}}_{\text{VA}}^{2}$) was 0.33. The three predictors also significantly contributed to isometric MVC in AT (*F*(3,10) = 9.11, *P* = 0.003). However, when controlling for other predictors, only RT significantly predicted isometric MVC in AT (*t* (10) = 4.65, *P* = 0.001; ${\text{sr}}_{\text{VA}}^{2}=0.58$). In AT–Control, the predictors significantly contributed to isometric MVC (*F*(3,10) = 7.29, *P* = 0.007). When controlling for the other predictors, both VA (*t* (10) = 2.29, *P* = 0.045; ${\text{sr}}_{\text{VA}}^{2}=0.16$) and RT (*t*(10) = 2.98, *P* = 0.01; ${\text{sr}}_{\text{VA}}^{2}=0.28$) were significant predictors of MVC torque in AT–Controls.

## DISCUSSION

This study is unique in that it evaluates neural and muscular contributions to maximal strength in AT. Despite similar plantar flexor strength, the contributions differed between AT and controls. In AT, maximal isometric torque was associated with RT, indicating contractile function predicted strength. In controls, VA (neural drive) was the largest predictor of maximal isometric torque. Systemic pain perception also differed: upper trapezius PPTs were elevated in AT.

There were no differences in maximal plantar flexion strength between AT and controls or between AT limbs. Although strength differences are commonly assumed in tendinopathy (1), a recent systematic review found conflicting evidence for impaired plantar flexor muscle strength in people with AT compared with controls (6). We showed that plantar flexor strength and power were not impaired at any velocity of contraction in people with AT.

RT is largely representative of contractile (muscular) mechanisms. However, the impact of tendon properties on electrically evoked torque amplitudes and differences in tendon properties between controls and those with AT may explain why maximal plantar flexor torque was best predicted by RT in AT (33). Furthermore, electrical stimulation intensities do not explain why neuromuscular mechanisms (i.e., VA and RT) contributed differently to maximal isometric strength.

That electromechanical delay was also not different between groups simply suggests that the onset of torque production is similar. The ultimate difference in RT could be the result of a reduced modulus of elasticity: with a smaller slope in the stress–strain relationship, a larger amount of tissue deformation would be required to produce similar levels of stress. These findings suggest that musculotendinous slowing may be responsible for differences in plantar flexor function in AT.

VA was the best predictor of isometric MVC torque in controls, echoing previous research evaluating plantar flexor function in young, healthy adults (31). However, VA had a lesser influence on isometric MVC torque with greater symptom severity: the proportional variance in isometric MVC uniquely explained by VA decreased from 0.33 in controls to 0.28 in AT–Controls to 0.01 in AT. In contrast, RT was the best predictor of isometric MVC torque in the more affected AT limb. RT played an increasingly prominent role in predicting isometric MVC as symptom severity increased: the proportional variance in isometric MVC that was uniquely explained by RT increased from 0.02 in controls to 0.28 in AT–Controls to 0.58 in AT.

Justas differences in RT are likely not explained by plantar flexor contractility, contractile mechanisms probably do not underlie the relationships between RT and MVC. If reductions in RT are indeed explained by impairments in tendon properties in AT, the impaired tendon properties could lessen the role of neural drive in predicting maximal strength and instead become the predominant predictor of maximal strength in AT.

Persons with AT were less sensitive to pain than controls in a region of the body not involved in the AT injury (i.e., the upper trapezius). These findings suggest a difference in systemic pain modulation. However, greater upper trapezius PPTs could reflect increased body weight and increased male participants in the AT group rather than meaningful differences in PPTs between AT and controls.

Previous research on pain sensitivity in AT is mixed. People with AT have been shown to have reduced PPTs (higher sensitivity), similar PPTs, and increased PPTs (lower sensitivity) when compared with controls (34–37). None of these studies limited their test group to midportion AT; they included insertional AT (34–37), persons with lateral ankle pain and heel pain (35), and persons with and without a history of tendon pain (37). The heterogeneity of findings suggests that AT may be too multifactorial to simplify into central versus peripheral processes.

The pain findings from PPTs, self-report measures, and clinical measures may seem conflicting at first glance; there were no differences in local PPTs (measured at the Achilles tendon), but there were group differences in self-reported pain and stiffness per VISA-A and FAAM, and palpation of the tendon resulted in pain reproduction in patients with AT. However, these outcome measures do not include “pain with palpation” among their list items. Instead, they include pain and stiffness in response to tendon loading activities, such as standing, walking, stair climbing, and participation in sport (23,24). Although pain is often reproducible with palpation of the tendon in AT, pressure applied to the tendon is not related to the mechanism of injury—the reason patients seek intervention—or the typical aggravating factors cited by patients with AT (3), and does not consistently correlate with symptoms (27). In addition, most control patients in this study reported pain during tendon palpation. This was unsurprising because even healthy tendons can be sensitive to pressure (3), yet it was neither a reproduction of previously experienced pain, nor did it accompany other positive findings suggestive of AT. Finally, PPTs were measured at a standardized site, rather than at the point of maximal tenderness to palpation, due to setup constraints from the Biodex footplate. Although these locations were similar (PPTs were measured at 4 cm, and average maximal pain was between 2.9 and 5.4 cm proximal), any variations from the point of maximal tenderness could have contributed to lesser sensitivity at the Achilles tendon than has been found in other studies.

### Translational Significance

Considering the magnitudes of muscle force observed in this study, resisted plantar flexion exercises using resistance bands are likely insufficient for engaging the Achilles tendon in AT. Considering an average internal moment arm of 5.2 cm at the talocrural joint, and an average MVC torque of 130.5 N·m, the average plantar flexor muscle force during isometric MVCs was approximately 2510 N. In contrast, the maximum forces produced by the stiffest therapeutic resistance bands at a 250% stretch range from 50 to 80 N (38). Even the stiffest resistance bands (80 N force) at this high level of prestretch would provide, at most, a force equivalent to 3.2% of isometric MVC force.

Rather than traditional muscle strengthening, the results of this study support approaching AT rehabilitation via a tendon loading paradigm. Exercise progression and dosage for muscle strengthening are well defined and relate to adaptations that occur within the muscle and nervous system (39,40). In contrast, parameters needed for an effective tendon-loading protocol in AT are largely undefined. Future research should focus on addressing this gap.

### Limitations

Neural and muscular mechanisms were investigated during isometric contractions only because the interpolated twitch technique is not well validated during dynamic contractions (41). Neural drive and muscular mechanisms may not behave identically during dynamic and isometric contractions. Finally, this study cannot attribute cause and effect to the differences in systemic pain modulation (elevated upper trapezius PPT) seen in persons with AT. Further research—and alternative study designs—would be required to establish causation.

### Conclusions

Plantar flexor strength and power differences cannot be assumed in AT, whether evaluated isometrically, concentrically, or eccentrically. Pain sensitivity quantified using algometry also cannot be assumed in AT. Despite similar magnitudes of RT and VA, their relationships to MVC differed between AT and controls. These findings suggest that prognosis in AT may not depend upon making gains in plantar flexor strength or power, a finding echoed by a recent systematic review (6). Instead, symptom resolution may be contingent upon normalizing function of the musculotendinous unit.

## Supplementary Material

Supplemental Figure 3, Resting Twitch Torque

Supplemental Figure 2, Normalized Isometric Torque

Supplemental Figure 1, Pressure-Pain Threshold Testing

## Figures and Tables

**Table 1:** Baseline Characteristics of the Test Groups.

Variable	Control	AT	*P* Value, Control vs AT	AT-Control	*P* Value, AT vs AT-Control

*n*	14 (7 male)	14 (10 male)	—	—	—
Age (yr)	23.6 (5.5)	31.0 (11.1)	0.04*	—	—
Height (m)	1.48 (0.11)	1.52 (0.09)	0.31	—	—
Weight (kg)	67.4 (13.0)	87.7 (21.3)	0.01*	—	—
BMI (kg⋅m^−2^)	22.9 (2.0)	28.5 (7.1)	0.01*	—	—
Fat mass (%)	24.1 (10.5)	27.5 (11.2)	0.41	—	—
Lean mass (%)	72.3 (9.3)	69.6 (10.6)	0.48	—	—
MAQ (MET⋅h⋅wk^−1^)	35.1 (26.2)	59.6 (53.6)	0.15	—	—
TSK	32.9 (6.9)	36.6 (9.7)	0.29	—	—
Symptom duration (mo)	0 (0.0)	66.9 (67.0)	0.002*		
FAAM-ADL (%)	99.2 (1.6)	82.1 (18.9)	0.005*	93.3 (10.9)	0.07
VISA-A (%)	99.2 (2.9)	63.0 (17.9)	<0.001*	78.1 (20.5)	0.048*
FAAM-Sport (%)	98.1 (3.0)	61.7 (16.7)	<0.001*	86.5 (14.2)	<0.001*
Isometric MVC (N⋅m)	113.0 (40.0)	121.1 (46.1)	0.89	144.1 (45.1)	0.19
RT amplitude (N⋅m)	34.9 (11.7)	34.6 (11.4)	0.07	38.8 (12.9)	0.37
VA (%)	80.2 (20.6)	81.2 (20.6)	0.53	88.2 (13.7)	0.30

Data presented as mean (standard deviation).

* Statistically significant (*P* < 0.05).

AT, midportion Achilles tendinopathy; BMI, body mass index; FAAM, Foot and Ankle Ability Measure, with Activities of Daily Living (ADL) and Sport subscales; MAQ, Modifiable Activity Questionnaire; TSK, Tampa Scale of Kinesiophobia; VA, voluntary activation; VISA-A, Victoria Institute of Sport Assessment-Achilles Questionnaire.

## Data Availability

The data sets generated and/or analyzed during the current study are available from the corresponding author upon reasonable request.
