# Supplementary figures and images for: Plantar Flexor Weakness and Pain Sensitivity Cannot Be Assumed in Midportion Achilles Tendinopathy
Source: Exerc Sport Mov. Author manuscript; Available in PMC 2024 Jan 12. (PMC10786321; doi:10.1249/esm.0000000000000017)

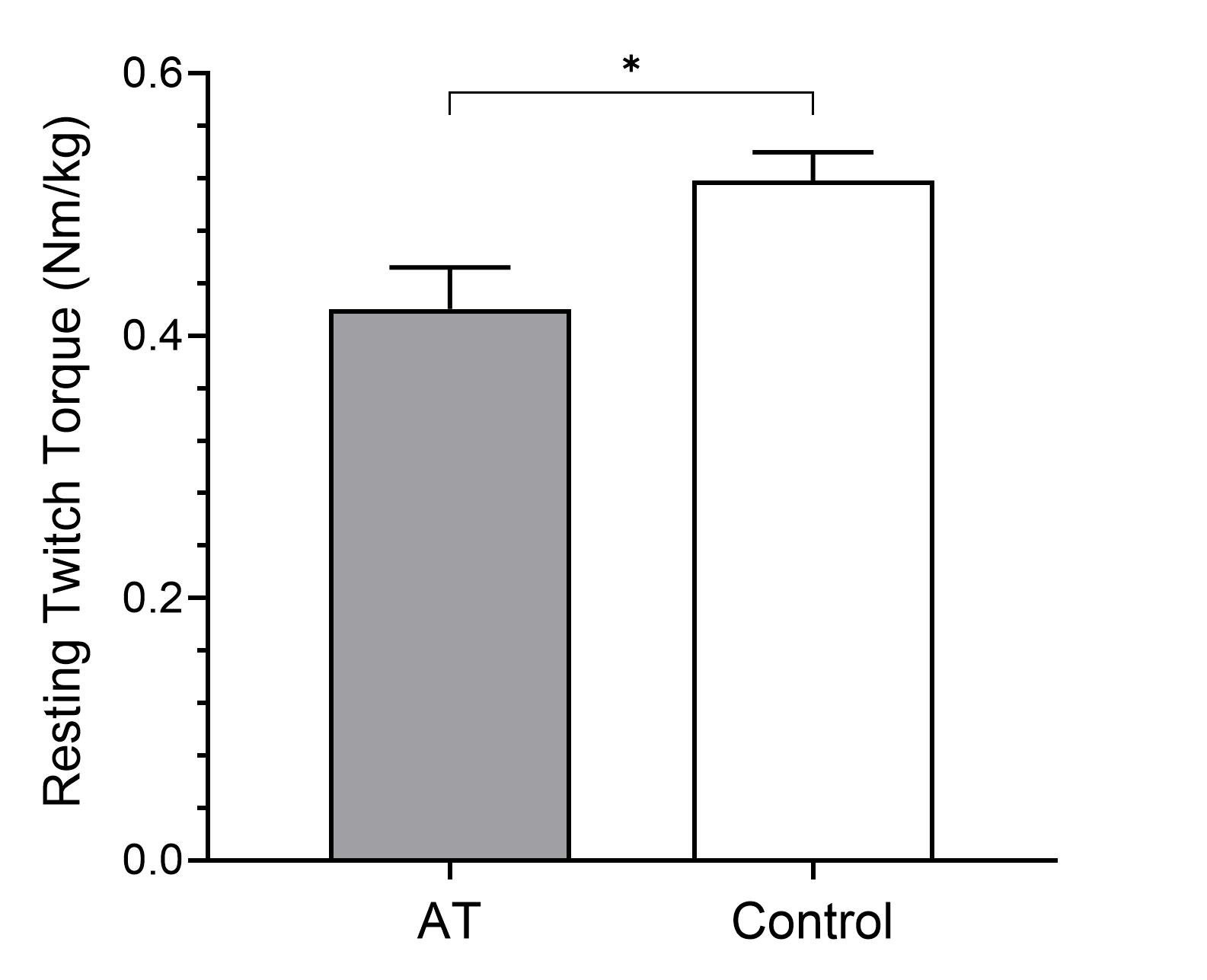

Supplement: Supplemental Figure 3, Resting Twitch Torque [file NIHMS1950686-supplement-Supplemental_Figure_3__Resting_Twitch_Torque.tif]

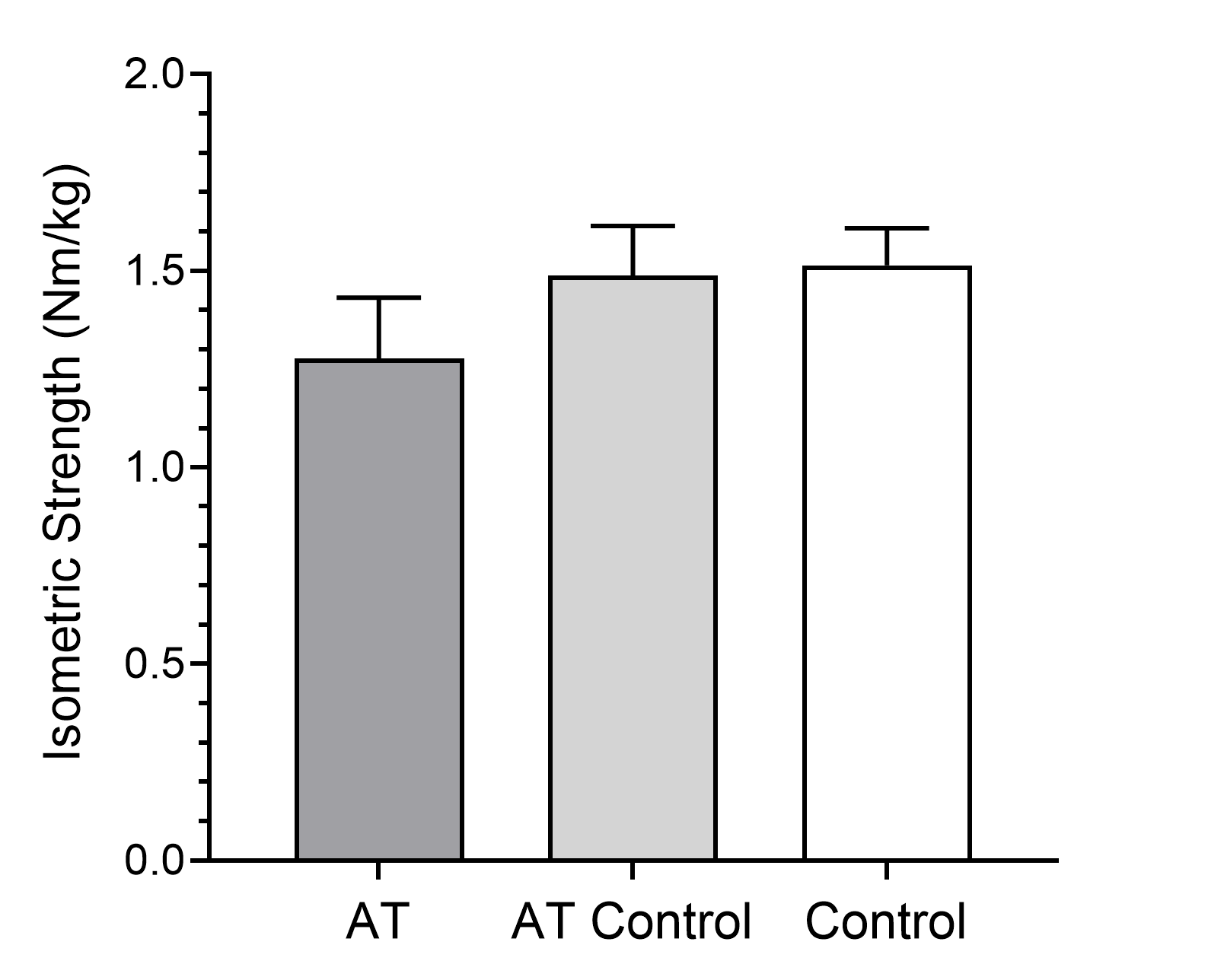

Supplement: Supplemental Figure 2, Normalized Isometric Torque [file NIHMS1950686-supplement-Supplemental_Figure_2__Normalized_Isometric_Torque.tif]

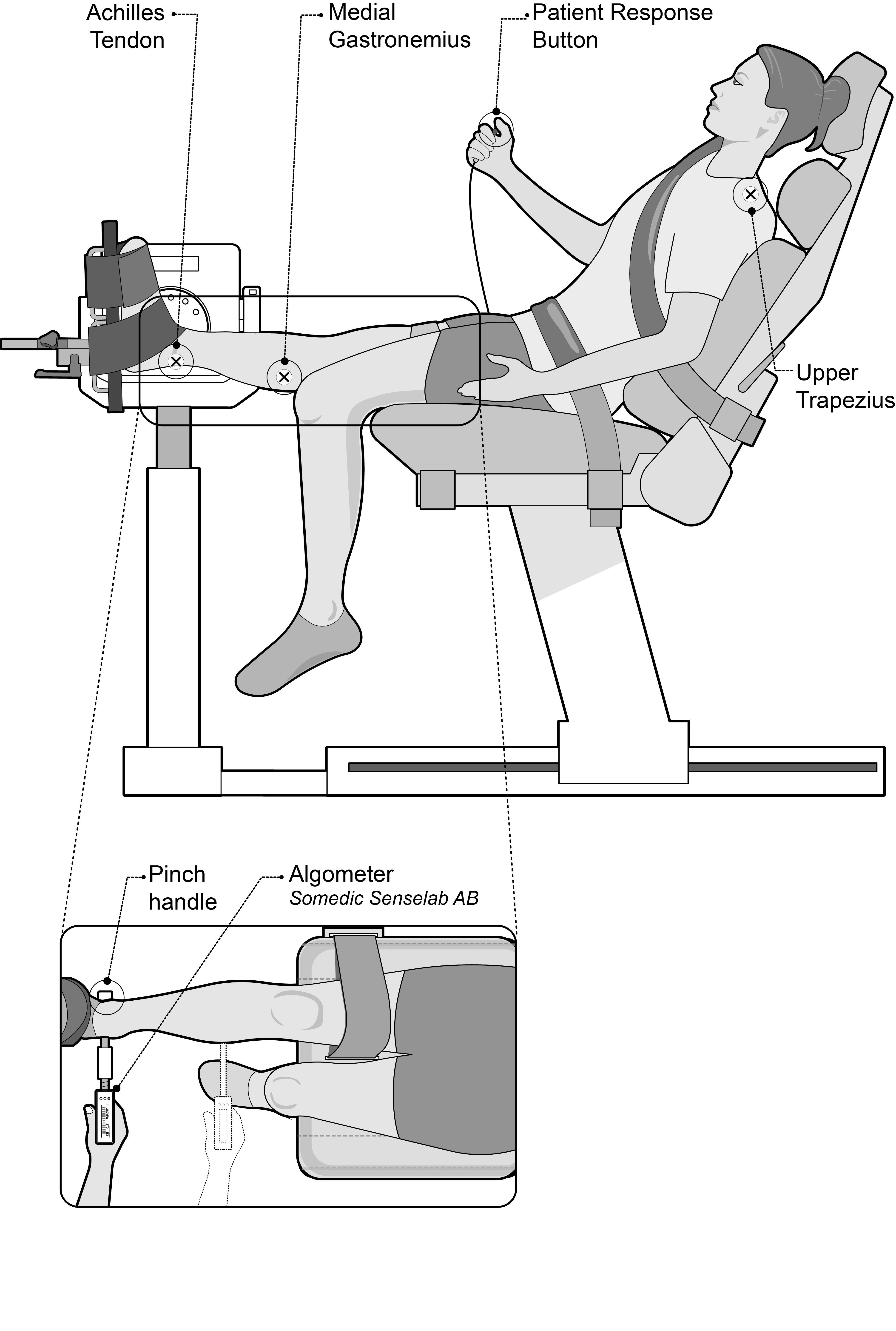

Supplement: Supplemental Figure 1, Pressure-Pain Threshold Testing [file NIHMS1950686-supplement-Supplemental_Figure_1__Pressure-Pain_Threshold_Testing.jpg]
